# Ten-year resistance trends in pathogens causing healthcare-associated infections; reflection of infection control interventions at a multi-hospital healthcare system in Saudi Arabia, 2007–2016

**DOI:** 10.1186/s13756-020-0678-0

**Published:** 2020-01-30

**Authors:** Hanan H. Balkhy, Aiman El-Saed, Majid M. Alshamrani, Asim Alsaedi, Wafa Al Nasser, Ayman El Gammal, Sameera M. Aljohany, Sara Almunif, Yassen Arabi, Saad Alqahtani, Henry Baffoe Bonnie, Majed Alghoribi, Adel Alothman, Saad A. Almohrij

**Affiliations:** 1 Infection Prevention and Control Department, King Abdulaziz Medical City (KAMC), Ministry of National Guard Health Affairs (MNGHA), Riyadh, Saudi Arabia; 2 King Saud bin Abdulaziz University for Health Sciences, Riyadh, Saudi Arabia; 3 King Abdullah International Medical Research Center, Riyadh, Saudi Arabia; 4 Community Medicine Department, Faculty of Medicine, Mansoura University, Mansoura, Egypt; 5 Infection Prevention and Control Department, KAMC, MNGHA, Jeddah, Saudi Arabia; 6 Infection Prevention and Control Department, Imam Abdulrahman bin Faisal Hospital, MNGHA, Dammam, Saudi Arabia; 7 Infection Prevention and Control Department, King Abdulaziz Hospital, MNGHA, Al hassa, Saudi Arabia; 8 Department of Pathology Medicine, KAMC, MNGHA, Riyadh, Saudi Arabia; 9 Department of Critical Care Medicine, KAMC, MNGHA, Riyadh, Saudi Arabia; 10 Department of Medicine, KAMC, MNGHA, Riyadh, Saudi Arabia; 11 Department of Surgery, KAMC, MNGHA, Riyadh, Saudi Arabia

**Keywords:** Antimicrobial resistance, Multidrug resistance, Healthcare-associated infections, Temporal trends, Hospital, Surveillance, Saudi Arabia

## Abstract

**Background:**

Studying temporal changes in resistant pathogens causing healthcare-associated infections (HAIs) is crucial in improving local antimicrobial and infection control practices. The objective was to describe ten-year trends of resistance in pathogens causing HAIs in a tertiary care setting in Saudi Arabia and to compare such trends with those of US National Health Surveillance Network (NHSN).

**Methods:**

Pooled analysis of surveillance data that were prospectively collected between 2007 and 2016 in four hospitals of Ministry of National Guard Health Affairs. Definitions and methodology of HAIs and antimicrobial resistance were based on NHSN. Consecutive NHSN reports were used for comparisons.

**Results:**

A total 1544 pathogens causing 1531 HAI events were included. Gram negative pathogens (GNP) were responsible for 63% of HAIs, with a significant increasing trend in *Klebsiella* spp. and a decreasing trend in *Acinetobacter*. Methicillin-resistant *Staphylococcus aureus* (27.0%) was consistently less frequent than NHSN. Vancomycin-resistant *Enterococci* (VRE, 20.3%) were more than doubled during the study, closing the gap with NHSN. Carbapenem resistance was highest with *Acinetobacter* (68.3%) and *Pseudomonas* (36.8%). Increasing trends of carbapenem resistance were highest in *Pseudomonas* and *Enterobacteriaceae*, closing initial gaps with NHSN. With the exception of *Klebsiella* and *Enterobacter*, multidrug-resistant (MDR) GNPs were generally decreasing, mainly due to the decreasing resistance towards cephalosporins, fluoroquinolones, and aminoglycosides.

**Conclusion:**

The findings showed increasing trends of carbapenem resistance and VRE, which may reflect heavy use of carbapenems and vancomycin. These findings may highlight the need for effective antimicrobial stewardship programs, including monitoring and feedback on antimicrobial use and resistance.

## Introduction

Healthcare-associated infections (HAIs) are associated with a considerable increase in morbidity, mortality, length of stay, disability, and healthcare cost [1–3]. These are believed to be further worsened by involvement of resistant pathogens, specially multi-drug resistant (MDR) ones [4–6]. Unfortunately, the contribution of resistant pathogens to HAIs is probably mounting in both increasing and decreasing infection settings [7, 8]. Studying the temporal changes of resistant pathogens responsible for documented HAIs is crucial in understanding the local epidemiology of HAI and improving local antimicrobial and infection control practices [9]. These trends are probably reflecting several interplaying practices/factors such as antimicrobial consumption [10, 11], infection control practices [12], environmental cleaning [13], and community burden [14].

The Infection Prevention and Control (IPC) department at Ministry of National Guard Health Affairs (MNGHA) Riyadh is serving as the hub for the Gulf Corporate Council (GCC) Center for Infection Control and the World Health Organization (WHO) Collaborating Center for IPC and antimicrobial resistance. The ICP department has been conducting focused surveillance in affiliated hospitals, based on the National Health Surveillance Network (NHSN) methods. Additionally, it has developed and is regularly updating a surveillance manual to standardize HAI definitions, data collection forms, and surveillance methods in the MNGHA hospitals and GCC member countries. Furthermore, surveillance data are centrally analyzed to provide regular standardized reports and publications. To date, the IPC department has published three benchmarking reports covering device-associated HAIs (DA-HAIs) in MNGHA and GCC hospitals [15–17]. Unfortunately, these reports were lacking information on resistance patterns of pathogens causing HAIs and their changes overtime. Moreover, such data are lacking regionally and limited internationally. The objective of the current study was to describe ten-year resistance trends in pathogens causing HAIs using the surveillance data collected from four MNGHA hospitals. Additionally, to compare such trends with corresponding trends in published NHSN report.

## Methods

### Setting

The surveillance datasets for four MNGHA affiliated hospitals were analyzed in this report. MNGHA hospitals are governmentally funded tertiary care hospitals that provide services for more than 1.5 million Saudi National Guard soldiers, employees and their families. The total bed capacity is 2200 beds with approximately 10% allocated for critical care services. Outpatient dialysis units include approximately 66 chairs used by more than 400 patients monthly. Approximately 30 thousands surgical procedures are performed in MNGHA hospitals every year. All hospitals are accredited by Joint Commission International (JCI). More details of MNGHA hospitals are shown in Additional file 1: Table S1.

### The ICP program at MNGHA hospitals

The ICP program is composed of an independent IPC department at each hospital that reports to the corporate ICP department in Riyadh. The latter ensure comparable practices and surveillance activities in all hospitals. The program is run by 24 infection preventionists; typically nurses with 2 years of infection training with or without CIBC certification (Additional file 1: Table S1). Each IPC department tailors its focused/targeted surveillance plan based on a local annual risk assessment that is approved by the IPC committee. Details of the IPC program activities to mitigate HAIs are shown in Additional file 1: Figure S1.

### Design

Pooled analysis of surveillance data that were prospectively collected between 2007 and 2016, using unified data collection forms and methods, adopted from the US NHSN [18].

### Infection and resistance definitions

HAIs included; central line-associated bloodstream infection (CLABSI), ventilator-associated pneumonia (VAP), catheter-associated urinary tract infections (CAUTI), dialysis access-related bloodstream infections (ARBSI), and surgical site infection (SSI). The surveillance definitions and data collection methods were based on the NHSN definitions, including the changes introduced in 2011 and 2013 [19]. While rare, more than one pathogen was allowed for a single HAI event. MDR definitions were retrospectively calculated as per the current NHSN definitions [20] and recent NHSN reports [21, 22]. Cephalosporin-resistant *Klebsiella* was defined as *Klebsiella* testing non-susceptible (resistant or intermediate) to at least one cephalosporin agent (ceftazidime, cefotaxime, ceftriaxone, or cefepime) [20]. Carbapenem-resistant *Enterobacteriaceae* (CRE) was defined as *Klebsiella*, *Escherichia coli*, or *Enterobacter* testing resistant to imipenem [20]. MDR gram negative pathogens (GNPs) were defined as pathogens testing non-susceptible (resistant or intermediate) to at least one agent in at least 3 out of 5 antimicrobial classes; aminoglycosides (amikacin or gentamicin), cephalosporins (ceftazidime, cefotaxime, ceftriaxone, or cefepime), fluoroquinolones (ciprofloxacin or levofloxacin), carbapenems (imipenem or meropenem), β-lactamase inhibitor (piperacillin or piperacillin/tazobactam) [21, 22]. Only in MDR *Pseudomonas*, 2 cephalosporins (cefepime and ceftazidime) rather than 4 cephalosporins (above) were considered.

### Event eligibility

All laboratory confirmed HAI events detected between 2007 and 2016 were initially included. Therefore, SSI, VAP, and neonatal “clinical sepsis” that were clinically diagnosed without laboratory confirmation were excluded. CAUTI events that are no longer meeting the latest definition (such as asymptomatic bacteriuria and fungal CAUTI) were excluded to allow more relevance of the study findings to current practices. Finally, HAI events lacking pathogen information were also excluded.

### Statistical methods

Categorical variables were presented as frequencies and percentages while continuous variables were presented as means and standard deviations. Age and gender were calculated for non-duplicate patients only. The distribution of pathogens and their resistance were presented overtime. The difference was examined using Mantel Haenszel Chi Square for linear trend. Two-year periods rather than one-year was chosen to allow for bigger number of events and consequently more reliable percentages. Trends of pathogen distribution and resistance were compared to corresponding trends in NHSN hospitals [21, 22]. HAI data in NHSN reports were combined and averaged per our study assigned time periods. Since 2007–2008 NHSN report provided only percentages, the average of all HAI events could not be estimated and was replaced by CLABSI percentages. SPSS (Version 25.0. Armonk, NY: IBM Corp) was used for all statistical analyses.

## Results

### HAI events and patients

Out of 2012 pathogens, 1544 pathogens linked to 1531 HAI events in 1333 patients were included. A total of 468 pathogens from 465 HAI events were excluded. These included 326 HAIs clinically diagnosed without laboratory confirmation; 197 SSI, 111 VAP, and 18 neonatal “clinical sepsis”. Additionally, 102 CAUTI with older criteria of diagnosis and 38 device-associated HAI with missing microbiological data were excluded. Details of included HAIs by hospital and hospital locations are shown in Additional file 1: Table S1. Approximately 52.8% of patients with included pathogens were females and the average age was 43.4 ± 27.0 years (79.1% adult patients and 20.9% pediatric/neonatal patients).

### Trends of causative pathogens

As shown in Table 1, GNPs were the most common (63.0%), followed by gram positive-pathogens (GPPs, 31.6%) and fungi (5.4%). The ranking in a decreasing order were: *Pseudomonas* (15.4%), *Klebsiella* (14.7%), *Staphylococcus aureus* (13.9%), *Enterobacter* (9.1%), and lastly *Escherichia coli* (9.1%). Among all pathogens, *Klebsiella* was the only pathogen to show a significant increasing trend during the study periods (*p*-value for trend = 0.016) while *Acinetobacter* was the only pathogen to show a significant decreasing trend during the study periods (*p*-value for trend = 0.009). The other pathogens tended to be stable during the study periods. *Enterobacter* and *Escherichia coli* showed slight but non-significant increase by the end of the study (2.2 and 1.3%, respectively) while *Enterococcus* and *Coagulase negative staphylococci* showed slight but non-significant decrease by the end of the study (− 1.6% and − 1.4%, respectively).

**Table 1:** Trends of distribution and rank order of pathogens causing healthcare-associated infections in four MNGHA hospitals in Saudi Arabia (2007–2016)

	2007–2008 *N* = 173		2009–2010 *N* = 427		2011–2012 *N* = 324		2013–2014 *N* = 360		2015–2016 *N* = 260		Total *N* = 1544		*P*-value*
	N (%)	R	N (%)	R	N (%)	R	N (%)	R	N (%)	R	N (%)	R	
Gram positives	67 (33.5%)		166 (33.8%)		91 (25.5%)		148 (35.7%)		81 (28.0%)		553 (31.6%)		0.211
*Staphylococcus aureus*	32 (16.0%)	2	53 (10.8%)	2	46 (12.9%)	3	64 (15.5%)	3	48 (16.6%)	2	243 (13.9%)	3	0.151
*Enterococcus spp.*	17 (8.5%)	4	52 (10.6%)	5	25 (7.0%)	8	37 (8.9%)	5	20 (6.9%)	6	151 (8.6%)	6	0.195
*Coagulase negative staphylococci*	7 (3.5%)	10	48 (9.8%)	6	16 (4.5%)	10	37 (8.9%)	5	6 (2.1%)	12	114 (6.5%)	7	0.114
Other gram positives	11 (5.5%)	8	13 (2.6%)	12	4 (1.1%)	14	10 (2.4%)	11	7 (2.4%)	11	45 (2.6%)	11	0.097
Gram negatives	125 (62.5%)		297 (60.5%)		232 (65.0%)		253 (61.1%)		196 (67.8%)		1103 (63.0%)		0.208
*Acinetobacter* spp.	14 (7.0%)	7	36 (7.3%)	7	19 (5.3%)	9	15 (3.6%)	8	12 (4.2%)	8	96 (5.5%)	8	0.009
*Pseudomonas* spp.	34 (17.0%)	1	70 (14.3%)	1	52 (14.6%)	2	65 (15.7%)	2	48 (16.6%)	2	269 (15.4%)	1	0.814
*Klebsiella* spp.	25 (12.5%)	3	53 (10.8%)	2	63 (17.6%)	1	68 (16.4%)	1	49 (17.0%)	1	258 (14.7%)	2	0.016
*Enterobacter* spp.	15 (7.5%)	6	53 (10.8%)	2	32 (9.0%)	6	32 (7.7%)	7	28 (9.7%)	4	160 (9.1%)	4	0.695
*Escherichia coli*	16 (8.0%)	5	35 (7.1%)	8	40 (11.2%)	4	41 (9.9%)	4	27 (9.3%)	5	159 (9.1%)	5	0.265
*Serratia* spp.	3 (1.5%)	13	13 (2.6%)	12	6 (1.7%)	12	13 (3.1%)	9	5 (1.7%)	13	40 (2.3%)	13	0.901
*Stenotrophomonas maltophia*	3 (1.5%)	13	17 (3.5%)	10	5 (1.4%)	13	9 (2.2%)	12	9 (3.1%)	10	43 (2.5%)	12	0.905
*Proteus* spp.	5 (2.5%)	12	4 (0.8%)	14	5 (1.4%)	13	3 (0.7%)	14	4 (1.4%)	14	21 (1.2%)	14	0.496
Other gram negatives	10 (5.0%)	9	16 (3.3%)	11	10 (2.8%)	11	7 (1.7%)	13	14 (4.8%)	7	57 (3.3%)	10	0.605
Fungi	8 (4.0%)		28 (5.7%)		34 (9.5%)	5	13 (3.1%)		12 (4.2%)		95 (5.4%)		0.259
*Candida* Spp.	7 (3.5%)	10	27 (5.5%)	9	32 (9.0%)	6	13 (3.1%)	9	10 (3.5%)	9	89 (5.1%)	9	0.225
Non-Candidal yeast	1 (0.5%)	15	1 (0.2%)	15	2 (0.6%)	15			2 (0.7%)	15	6 (0.3%)	15	0.876

*Abbreviations*: *N(%)* Number of pathogens and percentage, *R* Rank

* Mantel Haenszel Chi Square for linear trend

### Trends of resistant pathogens

The trends of antimicrobial resistance in different pathogens overtime are shown in Table 2. Overall, approximately 25% of both GPPs and GNPs had some type of resistance during the study. The most resistant pathogens were MDR *Stenotrophomonas* (70.0%), MDR *Acinetobacter* (64.1%), cephalosporin-resistant *Klebsiella* (32.1%), and methicillin-resistant *Staphylococcus aureus* (MRSA, 27.0%). CRE was significantly increasing from 0.0 to 11.4% (*p*-value for trend = 0.004). This was statistically evident in carbapenem-resistant *Escherichia coli* from 0.0 to 12.5% (*p*-value for trend = 0.007) and to less extent in carbapenem-resistant *Klebsiella* from 0.0 to 15.4% (*p*-value for trend = 0.066).

**Table 2:** Trends of antimicrobial resistance in selected pathogens causing healthcare-associated infections in four MNGHA hospitals in Saudi Arabia (2007–2016)

	2007–2008 *N* = 159			2009–2010 *N* = 382			2011–2012 *N* = 288			2013–2014 *N* = 344			2015–2016 *N* = 246			Total *N* = 1419			*P*-value*
	N	T (%)	R (%)	N	T (%)	R (%)	N	T (%)	R (%)	N	T (%)	R (%)	N	T (%)	R (%)	N	T (%)	R (%)	
Gram positives																			
MRSA	32	81%	30.8%	53	89%	14.9%	46	96%	29.5%	64	97%	32.3%	48	98%	27.7%	243	93.0%	27.0%	0.384
VRE	17	94%	6.3%	52	87%	11.1%	25	76%	52.6%	37	86%	21.9%	20	80%	18.8%	151	84.8%	20.3%	0.141
Overall resistance	49	86%	21.4%	104	88%	13.2%	70	90%	36.5%	97	94%	29.7%	67	93%	25.8%	387	90.2%	24.9%	0.071
Gram negatives																			
CephR *Klebsiella*	25	100%	24.0%	53	85%	42.2%	63	70%	31.8%	68	90%	27.9%	49	100%	32.7%	258	86.8%	32.1%	0.779
CRE *Klebsiella*	25	100%	0.0%	53	91%	8.3%	63	70%	6.8%	68	69%	8.5%	49	80%	15.4%	258	78.7%	8.4%	0.066
CRE *Enterobacter*	15	93%	0.0%	53	91%	2.1%	32	53%	0.0%	32	56%	0.0%	28	61%	0.0%	160	71.3%	0.9%	0.851
CRE E.Coli	16	100%	0.0%	35	97%	0.0%	40	78%	0.0%	41	76%	3.2%	27	89%	12.5%	159	85.5%	2.9%	0.007
Overall *Escherichia coli*	55	100%	0.0%	138	93%	3.9%	128	68%	3.4%	139	68%	5.3%	103	77%	11.4%	563	78.9%	5.0%	0.004
MDR *Acinetobacter*	14	100%	71.4%	36	94%	58.8%	19	95%	77.8%	15	100%	60.0%	12	92%	54.5%	96	95.8%	64.1%	0.665
MDR Pseudomonas*	34	100%	11.8%	70	97%	16.2%	52	98%	11.8%	65	98%	10.9%	48	98%	8.5%	269	98.1%	12.1%	0.353
MDR *Klebsiella*	25	100%	8.0%	53	85%	35.6%	63	78%	22.4%	68	85%	17.2%	49	94%	23.9%	258	86.4%	22.4%	0.967
MDR *Enterobacter*	15	87%	0.0%	53	53%	3.6%	32	38%	16.7%	32	41%	0.0%	28	54%	13.3%	160	50.6%	6.2%	0.248
MDR *Escherichia coli*	16	94%	26.7%	35	74%	34.6%	40	80%	18.8%	41	90%	18.9%	27	96%	19.2%	159	85.5%	22.8%	0.231
MDR *Serratia*	3	100%	33.3%	13	15%	0.0%	6	67%	25.0%	13	38%	0.0%	5	40%	0.0%	40	40.0%	12.5%	0.275
MDR *Stenotrophomonas*	3	100%	100.0%	17	24%	75.0%	5	0%		9	11%	0.0%	9	22%	50.0%	43	23.3%	70.0%	0.242
Overall resistance	102	100%	26.5%	254	89%	29.2%	199	85%	27.1%	222	89%	19.7%	167	87%	23.3%	944	89.2%	25.2%	0.085

*Abbreviations*: *N* Number of pathogens causing infection, *T (%)* Number of pathogens tested, *R (%)* Number of pathogens resistant, *MRSA* Methicillin-resistant *Staphylococcus aureus*, *VRE* Vancomycin-resistant *Enterococcus*, *CephR Klebsiella* Cephalosporin resistant *Klebsiella*, *CRE* Carbapenem resistant *Enterobacteriaceae*, *MDR* Multidrug resistant gram negative pathogens that tested non-susceptible (resistant or intermediate) to at least one agent in at least 3 out of 5 antimicrobial classes (see Methods). Overall resistance; an pathogen with one or more of the above types of resistance. * Mantel Haenszel Chi Square for linear trend

**Fig. 1:**
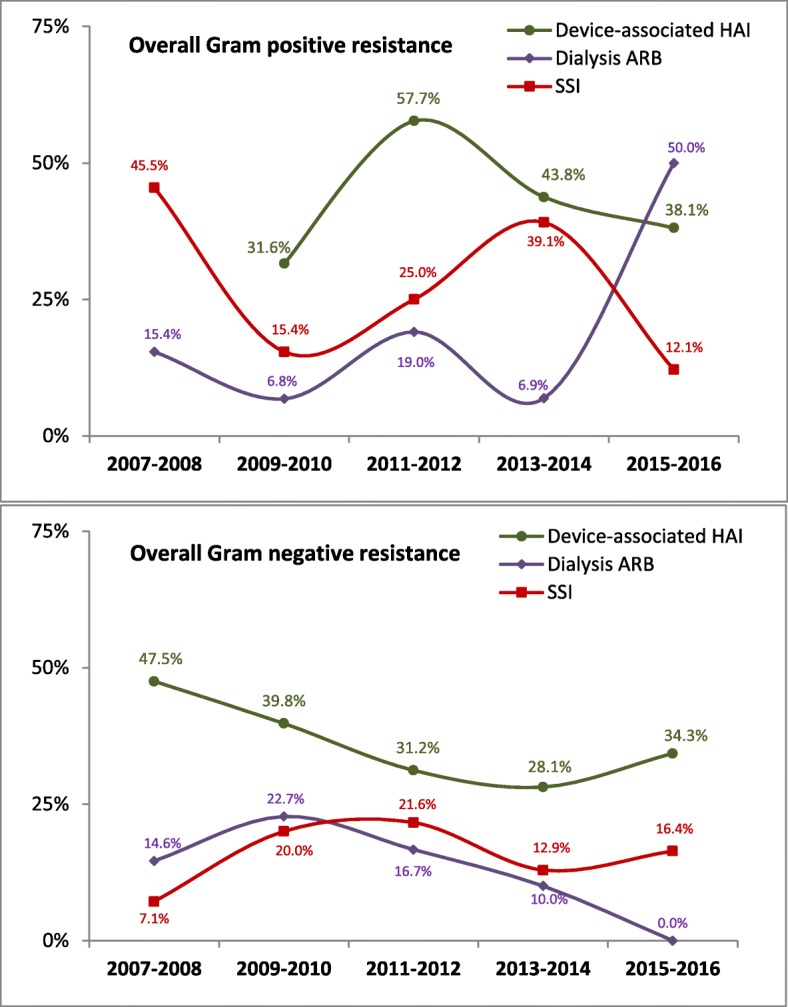
Trends of overall resistance of pathogens causing healthcare-associated infections by type of infection in four MNGHA hospitals in Saudi Arabia (2007–2016). Note: Gram positive resistance includes MRSA or VRE. Gram negative resistance include CephR *Klebsiella*, CRE, MDR *Acinetobacter*, MDR *Pseudomonas*, MDR *Klebsiella*, MDR *Escherichia coli*, MDR *Serratia*, or MDR *Stenotrophomonas*, as shown in Table 2. Device-associated HAI included central line–associated bloodstream infection, ventilator-associated pneumonia, and catheter-associated urinary tract infection

Overall GPP and GNP resistance by the type of HAI are shown in Fig. 1. Device-associated HAIs were presented as one group, as the small number of VAP and CAUTI did not allow breaking done the trends by the type of HAI and organisms combined. GPP resistance showed big variations overtime with a generally increased resistance in dialysis ARBSI and decreased resistance in SSI; none of which was statistically significant. On the other hand, GNP resistance showed a slight decreased resistance in device-associated HAI and dialysis ARBSI, also none were statistically significance (0.066 and 0.084, respectively).

More details regarding the trends of resistance for specific antimicrobial classes in GNPs are provided in Table 3 . For all GNPs combined, there were relative decreases in the resistance against aminoglycosides (31.6%), cephalosporins (26.4%), fluoroquinolones (16.6%), and b-lactam (12.2%) but 82.7% relative increase in the resistance against carbapenems by the end of the study. Carbapenem resistance was highest with *Acinetobacter* (68.3%) and *Pseudomonas* (36.8%). The increase in carbapenem resistance was highest in *Pseudomonas* (2.5 folds increase), increasing in *Klebsiella* and *Escherichia coli* (from zero to 33.3 and 15.8%, respectively), and non-existent in *Enterobacter. Klebsiella* was the only pathogen to show an increased resistance against all tested classes while *Acinetobacter* was the only pathogen to show a decreased resistance against all tested classes.

**Table 3:** Trends of resistance to specific antimicrobial classes in gram negative pathogens causing healthcare-associated infections in four MNGHA hospitals in Saudi Arabia (2007–2016)

	2007–2008 *N* = 113	2009–2010 *N* = 268	2011–2012 *N* = 212	2013–2014 *N* = 232	2015–2016 *N* = 180	Total *N* = 1005	Change*	
							Absolute	Relative
*Acinetobacter*								
Aminoglycosides	10 (71.4%)	16 (45.7%)	8 (44.4%)	7 (46.7%)	6 (50.0%)	47 (50.0%)	−21.4%	−30.0%
B-lactam	10 (76.9%)	14 (58.3%)	10 (90.9%)	6 (54.5%)	6 (75.0%)	46 (68.7%)	−1.9%	−2.5%
Carbapenems	10 (71.4%)	17 (63.0%)	13 (81.3%)	10 (66.7%)	6 (60.0%)	56 (68.3%)	−11.4%	− 16.0%
Cephalosporins	11 (84.6%)	22 (66.7%)	14 (82.4%)	13 (100.0%)	7 (70.0%)	67 (77.9%)	−14.6%	−17.3%
Fluoroquinolones	10 (71.4%)	20 (58.8%)	14 (77.8%)	10 (66.7%)	7 (63.6%)	61 (66.3%)	−7.8%	−10.9%
MDR3	10 (71.4%)	20 (58.8%)	14 (77.8%)	9 (60.0%)	6 (54.5%)	59 (64.1%)	−16.9%	−23.6%
MDR4	10 (71.4%)	18 (52.9%)	11 (61.1%)	7 (46.7%)	6 (54.5%)	52 (56.5%)	−16.9%	−23.6%
MDR5	8 (57.1%)	7 (20.6%)	5 (27.8%)	4 (26.7%)	6 (54.5%)	30 (32.6%)	−2.6%	−4.5%
*Pseudomonas*								
Aminoglycosides	6 (18.2%)	10 (14.9%)	6 (11.8%)	9 (13.8%)	3 (6.3%)	34 (12.9%)	−11.9%	−65.6%
B-lactam	9 (30.0%)	15 (25.0%)	2 (5.4%)	19 (30.6%)	8 (18.2%)	53 (22.7%)	−11.8%	−39.4%
Carbapenems	5 (15.2%)	11 (42.3%)	8 (40.0%)	9 (50.0%)	9 (52.9%)	42 (36.8%)	37.8%	249.4%
Cephalosporins*	12 (35.3%)	24 (36.4%)	6 (12.8%)	9 (15.0%)	4 (9.3%)	55 (22.0%)	−26.0%	−73.6%
Fluoroquinolones	2 (5.9%)	11 (16.4%)	7 (13.7%)	9 (14.8%)	5 (10.9%)	34 (13.1%)	5.0%	84.8%
MDR3	4 (11.8%)	11 (16.2%)	6 (11.8%)	7 (10.9%)	4 (8.5%)	32 (12.1%)	−3.3%	−27.7%
MDR4	2 (5.9%)	8 (11.8%)	4 (7.8%)	6 (9.4%)	2 (4.3%)	22 (8.3%)	−1.6%	−27.7%
MDR5	1 (2.9%)	4 (5.9%)	1 (2.0%)	5 (7.8%)	2 (4.3%)	13 (4.9%)	1.3%	44.7%
*Klebsiella*								
Aminoglycosides	7 (28.0%)	24 (46.2%)	20 (32.8%)	16 (24.6%)	15 (32.6%)	82 (32.9%)	4.6%	16.5%
B-lactam	3 (17.6%)	14 (50.0%)	10 (37.0%)	14 (31.8%)	15 (40.5%)	56 (36.6%)	22.9%	129.7%
Carbapenems	0 (0.0%)	4 (13.3%)	3 (11.5%)	4 (14.3%)	6 (33.3%)	17 (13.9%)	33.3%	–
Cephalosporins	6 (27.3%)	19 (63.3%)	14 (48.3%)	17 (34.7%)	16 (43.2%)	72 (43.1%)	16.0%	58.6%
Fluoroquinolones	4 (18.2%)	21 (46.7%)	19 (33.9%)	12 (18.5%)	11 (24.4%)	67 (28.8%)	6.3%	34.4%
MDR3	2 (8.0%)	16 (35.6%)	11 (22.4%)	10 (17.2%)	11 (23.9%)	50 (22.4%)	15.9%	198.9%
MDR4	2 (8.0%)	5 (11.1%)	3 (6.1%)	7 (12.1%)	7 (15.2%)	24 (10.8%)	7.2%	90.2%
MDR5	0 (0.0%)	1 (2.2%)	0 (0.0%)	2 (3.4%)	5 (10.9%)	8 (3.6%)	10.9%	–
*Enterobacte*								
Aminoglycosides	0 (0.0%)	7 (14.0%)	2 (6.7%)	5 (16.7%)	2 (7.1%)	16 (10.5%)	7.1%	–
B-lactam	2 (50.0%)	5 (22.7%)	3 (37.5%)	1 (50.0%)	2 (50.0%)	13 (32.5%)	0.0%	0.0%
Carbapenems	0 (0.0%)	1 (6.7%)	0 (0.0%)	0 (0.0%)	0 (0.0%)	1 (1.6%)	0.0%	–
Cephalosporins	2 (50.0%)	10 (52.6%)	3 (50.0%)	2 (33.3%)	5 (55.6%)	22 (50.0%)	5.6%	11.1%
Fluoroquinolones	0 (0.0%)	4 (8.7%)	2 (6.7%)	0 (0.0%)	3 (10.7%)	9 (6.1%)	10.7%	–
MDR3	0 (0.0%)	1 (3.6%)	2 (16.7%)	0 (0.0%)	2 (13.3%)	5 (6.2%)	13.3%	–
MDR4	0 (0.0%)	0 (0.0%)	1 (8.3%)	0 (0.0%)	0 (0.0%)	1 (1.2%)	0.0%	–
MDR5	0 (0.0%)	0 (0.0%)	0 (0.0%)	0 (0.0%)	0 (0.0%)	0 (0.0%)	0.0%	–
*Escherichia coli*								
Aminoglycosides	5 (31.3%)	14 (40.0%)	14 (35.0%)	12 (31.6%)	9 (34.6%)	54 (34.8%)	3.4%	10.8%
B-lactam	5 (45.5%)	6 (28.6%)	5 (35.7%)	6 (23.1%)	9 (60.0%)	31 (35.6%)	14.5%	32.0%
Carbapenems	0 (0.0%)	0 (0.0%)	0 (0.0%)	1 (4.8%)	3 (15.8%)	4 (4.4%)	15.8%	–
Cephalosporins	10 (66.7%)	10 (45.5%)	14 (56.0%)	17 (47.2%)	14 (53.8%)	65 (52.4%)	−12.8%	−19.2%
Fluoroquinolones	7 (46.7%)	13 (38.2%)	18 (51.4%)	18 (46.2%)	9 (36.0%)	65 (43.9%)	−10.7%	−22.9%
MDR3	4 (26.7%)	9 (34.6%)	6 (18.8%)	7 (18.9%)	5 (19.2%)	31 (22.8%)	−7.4%	−27.9%
MDR4	3 (20.0%)	1 (3.8%)	2 (6.3%)	3 (8.1%)	4 (15.4%)	13 (9.6%)	−4.6%	−23.1%
MDR5	0 (0.0%)	0 (0.0%)	0 (0.0%)	1 (2.7%)	0 (0.0%)	1 (0.7%)	0.0%	–
Others*								
Aminoglycosides	5 (50.0%)	7 (25.9%)	1 (11.1%)	4 (23.5%)	2 (16.7%)	19 (25.3%)	−33.3%	−66.7%
B-lactam	3 (42.9%)	2 (25.0%)	1 (33.3%)	2 (28.6%)	2 (28.6%)	10 (31.3%)	−14.3%	−33.3%
Carbapenems	3 (33.3%)	9 (56.3%)	2 (50.0%)	2 (40.0%)	7 (70.0%)	23 (52.3%)	36.7%	110.0%
Cephalosporins	5 (55.6%)	10 (52.6%)	2 (33.3%)	2 (22.2%)	4 (40.0%)	23 (43.4%)	−15.6%	−28.0%
Fluoroquinolones	2 (25.0%)	7 (25.0%)	1 (11.1%)	3 (16.7%)	1 (9.1%)	14 (18.9%)	−15.9%	−63.6%
MDR3	4 (40.0%)	5 (29.4%)	1 (16.7%)	1 (9.1%)	2 (22.2%)	13 (24.5%)	−17.8%	−44.4%
MDR4	2 (20.0%)	2 (11.8%)	0 (0.0%)	1 (9.1%)	1 (11.1%)	6 (11.3%)	−8.9%	−44.4%
MDR5	2 (20.0%)	2 (11.8%)	0 (0.0%)	0 (0.0%)	1 (11.1%)	5 (9.4%)	−8.9%	−44.4%
Overall								
Aminoglycosides	32 (31.7%)	75 (31.5%)	48 (25.4%)	50 (23.9%)	34 (21.7%)	239 (26.7%)	−10.0%	−31.6%
B-lactam	31 (41.9%)	54 (36.7%)	30 (33.0%)	45 (31.5%)	39 (36.8%)	199 (35.5%)	−5.1%	−12.2%
Carbapenems	18 (19.4%)	39 (32.2%)	26 (28.3%)	25 (26.3%)	29 (35.4%)	137 (28.4%)	16.0%	82.7%
Cephalosporins	48 (54.5%)	93 (54.1%)	52 (42.6%)	60 (37.0%)	51 (40.2%)	304 (45.3%)	−14.4%	−26.4%
Fluoroquinolones	25 (26.0%)	73 (32.3%)	59 (32.8%)	48 (23.3%)	33 (21.7%)	238 (27.7%)	−4.3%	−16.6%
MDR3	23 (23.0%)	61 (31.4%)	39 (25.5%)	33 (18.4%)	28 (20.1%)	184 (24.1%)	−2.9%	−12.4%
MDR4	19 (19.0%)	34 (17.5%)	21 (13.7%)	23 (12.8%)	19 (13.7%)	116 (15.2%)	−5.3%	−28.1%
MDR5	11 (11.0%)	12 (6.2%)	6 (3.9%)	12 (6.7%)	12 (8.6%)	53 (6.9%)	−2.4%	− 21.5%

*Abbreviations*: Others include *Serratia* spp., *Stenotrophomonas maltophia*, *Citrobacter* spp., *Proteus*, and *Providencia*. MDR3, MDR4, MDR5 are multidrug resistant gram negative pathogens that non-susceptible (resistant or intermediate) to at least one agent in at least 3, 4, or 5 out of 5 antimicrobial classes (respectively). *Absolute change is the difference between 2015 and 2016 rate and 2007–2008 rate. Relative change is the proportion of absolute change relative to 2007–2008 rate

### Comparisons with NHSN trends of resistant pathogens

**Fig. 2:**
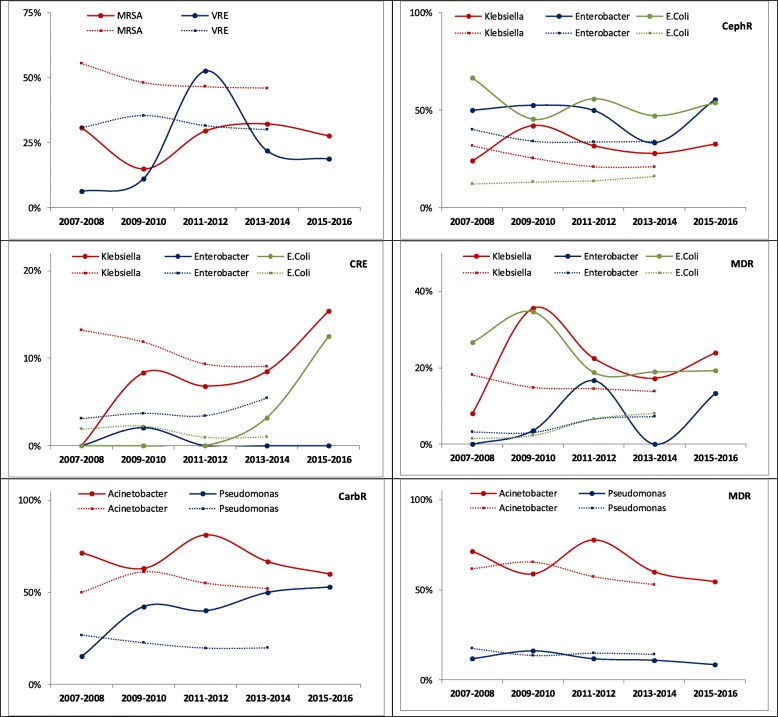
Trends of antimicrobial resistance in pathogens causing healthcare-associated infections in in MNGHA hospitals (2007–2015) and NHSN hospitals (2007–2014). Abbreviations: As in Table 2. CephR, cephalosporin resistant; CarbR, carbapenem resistant. Solid lines represent pathogens causing all HAI in MNGHA while dotted lines represents pathogens causing CLABSI, VAP, CAUTI, and SSI compiled from the NHSN reports

The trends of antimicrobial resistance in MNGHA compared with NHSN hospitals are shown in Fig. 2. The NHSN data points are up to year 2014 due to a lack of updated NHSN publication. MRSA was consistently less frequent in MNGHA than NHSN hospitals. VRE was more than doubled in MNGHA during the study closing the gap with NHSN hospitals. In *Escherichia coli* and *Klebsiella*, carbapenem resistance was increasing in MNGHA closing initial gaps with NHSN hospitals while cephalosporin-resistance and MDR were generally higher in MNGHA compared with NHSN hospitals. In *Acinetobacter* and *Pseudomonas*, carbapenem resistance was generally higher while MDR was generally comparable in MNGHA compared with NHSN hospitals.

## Discussion

The current report showed 10-year trends of the distribution and resistance of pathogens causing the five most commonly surveyed HAIs. The most interesting finding in the current study was the increasing contribution and resistance of *Enterobacteriaceae*. For example, the contribution of *Enterobacteriaceae* to HAIs increased from 28 to 33% during the study with concomitant emergence of CRE from 0 to 5%. *Klebsiella* spp. was the major component of CRE. Historically, carbapenem-resistant *Klebsiella pneumoniae* (CRKP) started in the USA then spread to Israel and other Middle Eastern counties [23–25]. The first outbreak of CRKP in MNGHA facilities was documented in 2010 [26] and was caused by an outer membrane protein [27]. The active surveillance that was started in response to that outbreak could not eliminate the risk of CRKP which continued to be seen thereafter at a lower level. This may explain the big difference in CRE between MNGHA and NHSN at the beginning of the study and the gradual narrowing of that difference thereafter. Additionally, local and national efforts done in the USA to reduce the burden of CRKP/CRE at both hospital and community settings lead to a clear downtrend [28, 29]. With increasing trend of CRE at MNGHA, we may see flipping of the MNGHA traditional comparisons of CRE with the NHSN in the coming years. Several local/regional challenges may explain the worsening of CRKP in Saudi Arabia and probably across the Middle East; variability of resistance mechanisms [25, 30], large transfer of workforce and pilgrimage [31, 32], and immature ASP practices [33].

Carbapenem resistance in the current study was highest in *Acinetobacter* and *Pseudomonas*, which exceeded NHSN figures [21], may reflect the local heavy use of carbapenems, which has been recently documented [34]. Emergence of novel resistant strains and increasing prevalence of high-risk clones has been suggested to explain the increasing carbapenem resistance in *Pseudomonas* in Saudi Arabia and GCC region [35]. Interestingly, *Acinetobacter* contribution to HAI and its carbapenem resistance were decreasing during the current study. The high *Acinetobacter* at the beginning of the study was caused by a documented outbreak of *Acinetobacter*-caused VAP [36, 37]. The outbreak triggered several interventions including a continuous active surveillance of *Acinetobacter* in MNGHA ICUs [36, 37].

With the exception of *Klebsiella* and *Enterobacter*, MDR gram negatives in the current study were generally decreasing, largely due to the decreasing resistance towards cephalosporins, fluoroquinolones, and aminoglycosides. Several efforts have been done in the last decade at MNGHA to reduce the burden of HAI and MDR; implementation of IHI preventive bundles for DA-HAIs [17, 38, 39], structuring/reinforcing a multifaceted hand hygiene program, gradual shifting from patient cohorting to single room isolation, and staff training and certification in infection control. However, the main challenge remains to further support and enhance the newly launched ASP. Major obstacles that are being currently managed include the transfer from paper to electronic medical records and limited ASP team members available for guidance and auditing, specially clinical pharmacists and infectious diseases physicians.

As expected, there was an increasing VRE trend but stable or slightly decreasing MRSA trend during the study (both did not reach significant levels through) [40, 41]. Traditional low rates of VRE in Saudi Arabia have been challenged in the last decade with increasing and novel resistance patterns [40, 42]. The increasing VRE trend during the study closed the gap initially observed between MNGHA and NHSN hospitals. These have been linked to extensive antimicrobial use in Saudi Arabia [43], including the ones on the top of the antimicrobial use list in our ICUs such vancomycin and piperacillin/tazobactam [34].

The current study was based on a large amount of data over a relatively long period of time which enabled us to monitor minor changes in antimicrobial resistance in several pathogens, which has not been matched in the region. The samples represented non-duplicate pathogens directly linked to the diagnosis of HAIs rather than unverified laboratory samples. Although a multi-hospital study, the data is considered very homogenous as the MNGHA hospitals share the same organizational structure, surveillance methodology, training, resources, and major related infection control interventions throughout the study period. For example, structuring/reinforcing multifaceted hand hygiene program, implementing the Institute for Healthcare Improvement (IHI) preventive bundles, and starting a stepwise antimicrobial stewardship program (ASP) all were done in comparable efforts during the same times. Nevertheless, the current data represented a scatter rather than comprehensive list of all HAIs during the study period due to two reasons; the targeted surveillance methodology and clinical diagnosis of some HAI. Yet, both were strictly done as per standard NHSN recommendations. Additionally, it would be better to have separate resistance trends for different HAIs. However, the small number of some HAIs made it impossible to break done the trends by the type of HAI and organisms combined. Finally, the changes in HAI definitions during the study may complicate the interpretation of data. However, this is an inherited limitation of any similar trend study and has been partially fixed by excluding diagnoses that are no longer acceptable such as CAUTI with only fungal pathogens.

## Conclusion

In conclusion, 10-year trends of pathogens causing commonly surveyed HAIs showed increasing contribution and resistance of *Enterobacteriaceae*. However, MDR gram negatives with the exception of *Klebsiella* and *Enterobacter* were generally decreasing. Carbapenem resistance was highest in *Acinetobacter* and *Pseudomonas*. There was an increasing VRE trend but stable or slightly decreasing MRSA trend during the study. The increasing trends of both CRE and VRE can be at least partially explained by the extensive use of broad-spectrum antimicrobials such as carbapenems, piperacillin/tazobactam, and vancomycin that has been recently documented in our patients [34]. These findings may highlight the need for effective antimicrobial stewardship programs, focusing on education, restrictions, monitoring, and feedback on antimicrobial use and resistance.

## Supplementary information

**Additional file 1: Table S1.** Characteristics of included hospitals* and healthcare-associated infections (2007–2016). **Figure S1.** Trends of overall resistance of pathogens causing healthcare-associated in relation to starting implementation of related infection control activities in four MNGHA hospitals in Saudi Arabia (2007–2016).

## Data Availability

Available upon request.
