# Supplementary material for: Ten-year resistance trends in pathogens causing healthcare-associated infections; reflection of infection control interventions at a multi-hospital healthcare system in Saudi Arabia, 2007–2016
Source: Antimicrob Resist Infect Control. 2020 Jan 30;9:21. doi: 10.1186/s13756-020-0678-0 (PMC6993320; doi:10.1186/s13756-020-0678-0)
Supplement: Supplementary file 1 — Additional file 1: Table S1. Characteristics of included hospitals* and healthcare-associated infections (2007–2016). Figure S1. Trends of overall resistance of pathogens causing healthcare-associated in relation to starting implementation of related infection control activities in four MNGHA hospitals in Saudi Arabia (2007–2016). [file 13756_2020_678_MOESM1_ESM.docx]

**Table S1 Characteristics of included hospitals*** and **healthcare-associated infections (2007-2016)**

|  | **KAMC-R** | **KAMC-J** | **KAH** | **IABFH** | **Overall** |
| --- | --- | --- | --- | --- | --- |
| Hospital statistics |  |  |  |  |  |
| Number of served military personnel and their families | 910,000 | 347,000 | 205,000 | 111,000 | 1,573,000 |
| Number of beds (including ICU beds) | 1000 (140) | 750 (51) | 293 (18) | 159 (6) | 2202 (215) |
| Admissions per year | 35,126 | 23,483 | 14,099 | 8,302 | 81,010 |
| Patient days per year | 305,445 | 160,880 | 73,417 | 25,625 | 565,367 |
| Average length of stay | 8.6 | 6.6 | 5.2 | 3.4 | 6.9 |
| Number of dialysis chairs | 38 | 18 | 10 | 0 | 66 |
| Surgical operations done per year | 14,507 | 10,935 | 4,110 | 2,328 | 31,880 |
| Infection control program statistics |  |  |  |  |  |
| Number of infection preventionists (certified) | 12 (6) | 6 (2) | 3 (2) | 3 (1) | 24 (11) |
| Number of infection preventionists (certified)/100 beds | 1.20 (0.60) | 0.80 (0.27) | 1.02 (0.68) | 1.89 (0.63) | 1.09 (0.50) |
| Number of infectious diseases physicians | 3 | 1 | 1 | 1 | 6 |
| Types of HAIs caused by the included pathogens |  |  |  |  |  |
| Central line–associated bloodstream infection (CLABSI) | 237 | 230 | 77 | 11 | 555 (36.3%) |
| Ventilator-associated pneumonia (VAP) | 9 | 22 | 24 | 7 | 62 (4.0%) |
| Catheter-associated urinary tract infection (CAUTI) | 21 | 18 | 15 | 9 | 63 (4.1%) |
| Dialysis access-related bloodstream infection (ARB) | 289 | 128 | 37 | 0 | 454 (29.7%) |
| Surgical site infection (SSI) | 173 | 148 | 20 | 56 | 397 (25.9%) |
| Total | 729 | 546 | 173 | 83 | 1531 (100.0%) |
| Location of HAIs caused by the included pathogens |  |  |  |  |  |
| Intensive care units | 365 | 138 | 120 | 24 | 647 (42.3%) |
| Wards | 75 | 280 | 16 | 59 | 430 (28.1%) |
| Outpatients | 289 | 128 | 37 | 0 | 454 (29.7%) |

* Hospital and infection control program statistics reflects the end of study (2016). KAMC-R, King Abdulaziz Medical City-Riyadh; KAMC-J, King Abdulaziz Medical City-Jeddah; KAH, King Abdulaziz Hospital-Alhassa; IABFH, Imam Abdulrahman Bin Faisal Hospital-Dammam; HAIs, Healthcare-associated infections

|  |
| --- |
|  |

**Figure S1: Trends of overall resistance of pathogens causing healthcare-associated in relation to starting implementation of related infection control activities in four MNGHA hospitals in Saudi Arabia (2007-2016)**

Note: Gram positive resistance includes MRSA or VRE. Gram negative resistance include CephR Klebsiella, CRE, MDR Acinetobacter, MDR Pseudomonas, MDR Klebsiella, MDR E-coli, MDR Serratia, or MDR Stenotrophomonas, as shown in Table 2. IPC, infection prevention and control; JCI, Joint Commission International; CRKP, Carbapenem-resistant Klebsiella pneumonia; ASP, antimicrobial stewardship program; GCC, Gulf Cooperation Council
